# Supplementary material for: Early correlates of visual awareness are affected by self-related information
Source: Neurosci Conscious. 2026 Jul 30;2026(1):niag042. doi: 10.1093/nc/niag042 (PMC13422636; doi:10.1093/nc/niag042)
Supplement: Supplementary_material_niag042 [file supplementary_material_niag042.zip › Supplementary_Material_Ciorlietal.docx]

Supplementary Material for

**Early Correlates of Visual Awareness Are Affected by Self-Related Information**

Ciorli, T.^a,*^, MacGregor, O.^b^, Nilsson, M.^c,d^, Borriero, A.^a^, Smate, I. ^c^, Kalckert, A.^c^, & Pia, L.^a,e^

^a^ Department of Psychology, University of Turin, Turin, Italy

^b^ Department of Information Technology, University of Skövde, School of Informatics, Skövde, Sweden

^c^ Department of Cognitive Neuroscience and Philosophy, University of Skövde, Skövde, Sweden

^d^ Department of Neurobiology, Care Sciences, and Society, Karolinska Institutet and Stockholm University, Stockholm, Sweden

^e^ Neuroscience Institute of Turin, Turin, Italy

**Supplementary Analyses**

*Localization and discrimination performance across masking*

Beside categorizing aware and unaware trials through PAS, we also analyzed behavioral performances of the localization and discrimination tasks considering masked and unmasked trials for awareness classification. First, we performed a 2 × 2 ANOVA with the factors Identity and Masking on localization *d′*. The only significant effect was the main effect of Masking (F_(1,35)_ = 44.46, *p* < .001), showing that localization performance was better in unmasked compared to masked trials despite stimulus identity (see **Fig**. **S1A**). As for the discrimination task, we could not compute identity-related *d’* in the conscious condition because of the absence of no-stimulus trials within the unmasked condition, so we computed identity-related discrimination *d’* only within the masked one. We then performed a t-test comparing discrimination *d*′ in masked trials between self and familiar faces, which was not significant, although a trend was observed (*p* = 0.067; see **Fig**. **S1B**). Overall, these analyses yield the same pattern of results when PAS scores are used as the awareness classifier.

Insert Supplementary Figure S1 here

*Distribution and mean PAS scores across masking condition as a function of face identity*

To investigate how trials were distributed across experimental conditions, we performed additional analyses and graphical representations of subjective awareness. Specifically, we analyzed the distribution of Perceptual Awareness Scale (PAS) ratings separately for masking condition (masked vs. unmasked) and Identity (Self vs. Familiar), considering both individual PAS scores (1–4; see **Fig. S2A**) and aggregated awareness categories (PAS 1–2 = unaware; PAS 3–4 = aware; see **Fig. S2C**). In addition, mean PAS scores across conditions were analyzed and plotted (see **Fig. S2B).** Because trial distributions (%) and mean PAS scores were not normally distributed, statistical comparisons were performed using Wilcoxon signed-rank tests, with false discovery rate (FDR) correction applied separately for each family of comparisons. These analyses were conducted to assess whether the amount of masked trials differed between Identity conditions across PAS levels, which could potentially influence VAN amplitude estimates. Specifically, a greater proportion of masked trials in one Identity condition could contribute to the differential VAN amplitudes observed in the main analyses.

Within masked trials, the percentage distribution of trials was comparable between the Self and Familiar conditions across both individual PAS scores (minimum *p* = .63; see **Fig**. **S2A**) and aggregated awareness categories (minimum *p* = .99; see **Fig. S2C**). Similar results were observed for unmasked trials (minimum *p* = .13). These findings indicate that Self and Familiar face trials were similarly distributed across PAS levels in both masked and unmasked conditions. Consistently, analyses of mean PAS ratings revealed no differences between Self and Familiar conditions in either masked or unmasked trials (minimum *p* = .24; see **Fig. S2B**), further suggesting comparable subjective awareness across identity conditions.

Importantly, a substantial proportion of masked trials was assigned to PAS 3 and, to a lesser extent, PAS 4 (**Fig. S2C**). This observation supports the methodological choice to classify aware and unaware trials primarily on the basis of subjective PAS ratings rather than masking condition alone, as masking per se did not perfectly predict subjective awareness. Although stimulus durations in masked trials were individually adjusted through a staircase procedure to achieve near-threshold perception, habituation effects and fluctuations in subjective sensitivity during the experiment may have altered awareness thresholds over time. Notably, these effects were not specific to either identity condition.

At the same time, the proportion of aware trials (PAS 3–4: 67.41 ± 14.81%) was substantially greater than that of unaware trials (PAS 1–2: 32.58 ± 13.88%), resulting in an unequal distribution of trial counts across awareness conditions. Although this imbalance may have influenced signal-to-noise ratio estimates, it was comparable between Self and Familiar conditions and therefore cannot account for the differential VAN effects observed between identities.

Insert Supplementary Figure S2 here
